# Intron-mediated enhancement of *DIACYLGLYCEROL ACYLTRANSFERASE*1 expression in energycane promotes a step change for lipid accumulation in vegetative tissues

**DOI:** 10.1186/s13068-023-02393-1

**Published:** 2023-10-14

**Authors:** Viet Dang Cao, Guangbin Luo, Shelby Korynta, Hui Liu, Yuanxue Liang, John Shanklin, Fredy Altpeter

**Affiliations:** 1 Agronomy Department, Plant Molecular and Cellular Biology Program, Genetics Institute, University of Florida, IFAS, Gainesville, FL USA; 2 DOE Center for Advanced Bioenergy and Bioproducts Innovation, Gainesville, FL USA; 3 Biology Department, Brookhaven National Laboratory, Upton, NY USA; 4 DOE Center for Advanced Bioenergy and Bioproducts Innovation, Upton, NY USA; 5 Biosciences Department, Brookhaven National Laboratory, Upton, NY USA

**Keywords:** Energycane, *WRINKLED*1, *DIACYLGLYCEROL ACYLTRANSFERASE*1, *OLEOSIN*1, Triacylglycerol, Metabolic engineering, Lipids, Biofuel, Bioenergy, Intron-mediated enhancement, Transgene expression

## Abstract

**Background:**

Metabolic engineering for hyperaccumulation of lipids in vegetative tissues is a novel strategy for enhancing energy density and biofuel production from biomass crops. Energycane is a prime feedstock for this approach due to its high biomass production and resilience under marginal conditions. *DIACYLGLYCEROL ACYLTRANSFERASE* (*DGAT*) catalyzes the last and only committed step in the biosynthesis of triacylglycerol (TAG) and can be a rate-limiting enzyme for the production of TAG.

**Results:**

In this study, we explored the effect of intron-mediated enhancement (IME) on the expression of *DGAT*1 and resulting accumulation of TAG and total fatty acid (TFA) in leaf and stem tissues of energycane. To maximize lipid accumulation these evaluations were carried out by co-expressing the lipogenic transcription factor *WRINKLED*1 (*WRI*1) and the TAG protect factor oleosin (*OLE*1). Including an intron in the codon-optimized *TmDGAT*1 elevated the accumulation of its transcript in leaves by seven times on average based on 5 transgenic lines for each construct. Plants with *WRI*1 (W), *DGAT*1 with intron (Di), and *OLE*1 (O) expression (WDiO) accumulated TAG up to a 3.85% of leaf dry weight (DW), a 192-fold increase compared to non-modified energycane (WT) and a 3.8-fold increase compared to the highest accumulation under the intron-less gene combination (WDO). This corresponded to TFA accumulation of up to 8.4% of leaf dry weight, a 2.8-fold or 6.1-fold increase compared to WDO or WT, respectively. Co-expression of WDiO resulted in stem accumulations of TAG up to 1.14% of DW or TFA up to 2.08% of DW that exceeded WT by 57-fold or 12-fold and WDO more than twofold, respectively. Constitutive expression of these lipogenic “push pull and protect” factors correlated with biomass reduction.

**Conclusions:**

Intron-mediated enhancement (IME) of the expression of *DGAT* resulted in a step change in lipid accumulation of energycane and confirmed that under our experimental conditions it is rate limiting for lipid accumulation. IME should be applied to other lipogenic factors and metabolic engineering strategies. The findings from this study may be valuable in developing a high biomass feedstock for commercial production of lipids and advanced biofuels.

**Graphical abstract:**

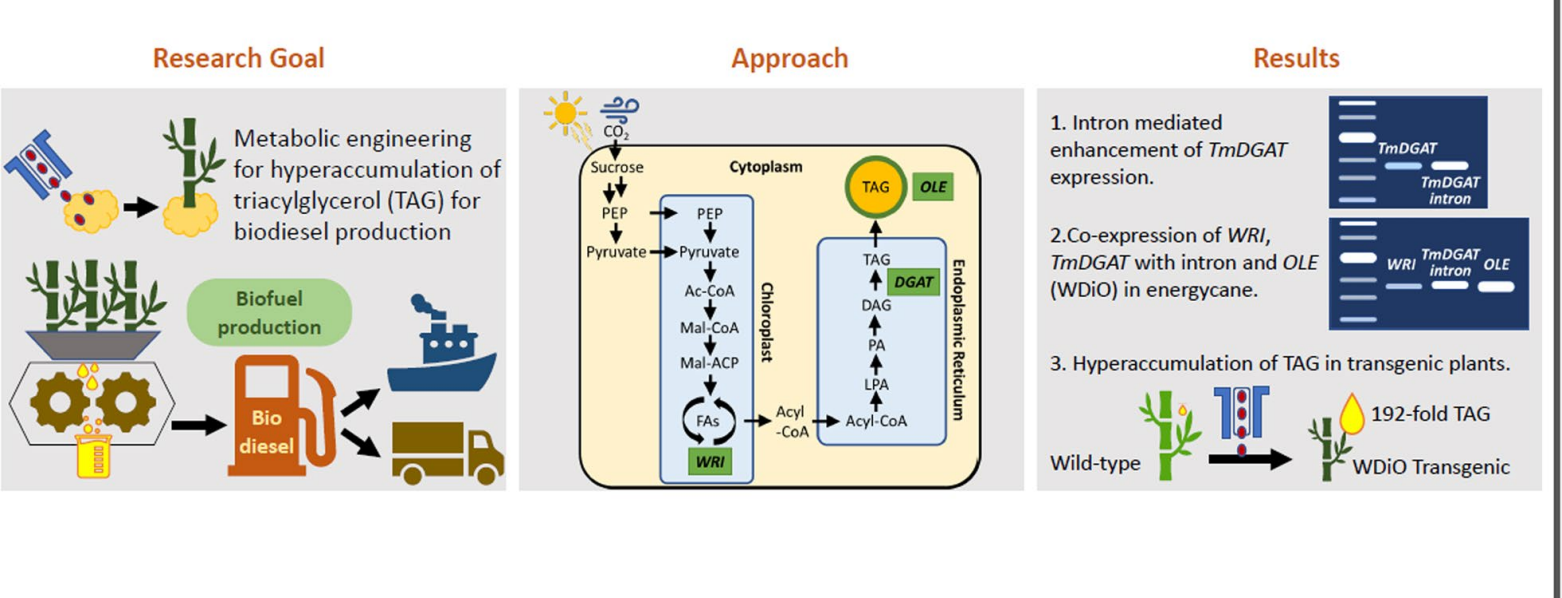

**Supplementary Information:**

The online version contains supplementary material available at 10.1186/s13068-023-02393-1.

## Background

Production of biofuel from perennial crops will increase energy security by reducing the need for fossil fuels while increasing carbon sequestration for combating climate change [1, 2]. Therefore, many countries have implemented government policies and subsidies promoting the production and consumption of bioenergy [3].

Energycane, is one of the most promising bioenergy feedstocks. Both energycane and sugarcane are highly polyploid, interspecific hybrids between *Saccharum officinarum* and *Saccharum spontaneum*, but energycane has a higher proportion of *Saccharum spontaneum* in its genome. This contributes to greater biomass yields, elevated resilience under marginal conditions, lower sugar, and higher fiber content in the stems of energycane relative to sugarcane [4–7]. Energycane’s superior resilience and biomass accumulation are supported by a more vigorous root system and higher light interception efficiency than sugarcane. The elevated light interception efficiency is associated with a higher tiller density and leaf inclination angles, contributing to the greatest photosynthetic efficiency among crops [8]. Photosynthesis, which transforms sunlight into stored chemical energy creates biomass that can be converted to renewable biofuel. Plant oil, consisting mainly of triacylglycerol [TAG; (98%)] and other fatty acids [9, 10] has more than twice the energy content of carbohydrates [11, 12]. Oil is typically produced from seeds of palm and oilseed crops such as soybean (*Glycine max*), sunflower (*Helianthus annus*) and canola (*Brassica napus*) [13]. Vegetative plant tissues including leaves, stems and roots are able to produce lipids and TAG in small quantities [14, 15]. The genes involved in the fatty acid and TAG synthesis, and hydrolysis have been identified [14, 16]. This enables metabolic engineering of high biomass crops including sugarcane and energycane for the hyperaccumulation of lipids in their vegetative biomass and has the potential to exceed oil yields of traditional oilseed crops [12, 17–20]. Ectopic expression of individual lipogenic factors led to modest lipid accumulation in vegetative tissues. A step change in accumulation of vegetative lipids has been reported following multigene engineering termed “push–pull-protect strategy” including genes involved in fatty acid biosynthesis (‘push’), TAG assembly (‘pull’), and suppression of lipid turnover (‘protect’) [21]. This approach optimized the flux of carbon into TAG at multiple metabolic levels, leading to hyperaccumulation of TAG in the vegetative tissues of model [22–25] and high biomass crops such as ryegrass [26], sugarcane [18, 20], sorghum [27], and potato [28]. *WRI*1 is a transcription factor of the family of APETALA2/ethylene-responsive element binding proteins involved in the regulation of fatty acid biosynthesis [29–33]. *DGAT*1 catalyzes the only committed step in the biosynthesis of TAG by catalyzing the transfer of an acyl group from acyl-CoA to diacylglycerol [34]. *DGAT*1 has been classified into three types; two endoplasmic reticulum (ER)-localized forms of *DGAT*1 (type-1) and *DGAT*2 (type-2) and a cytosolic form of the *DGAT* family (Type-3) [35, 36–38]. Overexpression of *DGAT*1 alone has been reported to increase the TAG content in the transgenic leaves by 20-fold compared to control [39]. Protection of TAG from turnover by lipases has been achieved by coating oil bodies with oleosin [22]. *OLEOSIN*1 (*OLE*1) is a structural protein that protects oil bodies from coalescing and reduces lipid breakdown by lipases [40, 41]. Previous research has shown increased fatty acid content of Arabidopsis vegetative tissues through the engineering of *OLE*1 (*CysOLE*1) by the substitution of six cysteine residues for their native equivalents within the N- and C-terminal hydrophilic domains [42].

We earlier reported that in the monocot *Lemna japonica,* co-expression of *WRI*1, *DGAT*1 and *OLE*1 had a much larger effect on TAG accumulation than co-expression of *WRI*1 and *OLE*1 without *DGAT*1, suggesting that the elevated FA synthesis due to ectopic *WRI*1 expression exceeds the capacity of the native *DGAT* to transfer FA to TAG [43]. Elevating the expression of *DGAT* is therefore a promising approach for hyperaccumulation of TAG in vegetative tissues. Various optimizations including stronger promoters or enhanced UTR’s, codon optimization or the presence of intron(s) have been described to elevate transgene expression in plants [44]. Introns can have a surprisingly large effect on gene expression, revealing a gap in our understanding of the molecular basis underlying this phenomenon [45–47].

In this study, we found that the addition of an intron from the predicted 5-methyltetrahydropteroyltriglutamate-homocysteine methyltransferase 1 of *Sorghum bicolor* into a codon-optimized *TmDAGT*1 from nasturtium dramatically increased the gene expression under control of the strong constitutive *Panicum virgatum* ubiquitin promoter (pPvUbiII). The elevated *TmDGAT*1 expression displayed strong synergy in TAG and TFA accumulation following the co-expression of *WRI*1, *DGAT*1, and *OLE*1 in energycane.

## Results

### *Cis*-regulatory elements in the 110-bp intron

A putative GT1 consensus and CAAT box were identified at the position of 52 bp and 93 bp, respectively, in the 110-bp-long intron that we inserted into *DGAT*1 and which originated from the predicted 5-methyltetrahydropteroyltriglutamate-homocysteine methyltransferase 1 of *Sorghum bicolor* (NCBI accession number: NC_012877.2) (Figure S1). Sequences with similarity to motifs that were earlier reported in IME including YATCN, the reverse complement of NGATY and similar sequence to TTNGATYTG and its reverse complement CAYATCNAA were also identified.

### Overexpression of *WRI*1, *DGAT*1 and *OLE*1 altered TAG and TFA production in leaves and stems of transgenic energycane

*WRI*1, *DGAT*1*,* and *OLE*1 expression was analyzed from leaf and stem tissues by qRT-PCR with primers shown in Table S1. To determine potential intron-mediated regulation of gene expression, we analyzed the expression in five PCR-positive lines for *DGAT*1 with intron (*DGAT*1 (In)) and compared this with the expression of in five PCR-positive lines for *DGAT*1 without intron (*DGAT*1 (W)). *DGAT*1 (In) elevated expression in leaves by seven times on average of 5 transgenic lines compared to *DGAT*1 (W) (Table 1). Expression in stem was two times higher for *DGAT*1 (In) compared to *DGAT*1 (W) (Table 2).

**Table 1:** Summary of TAG and TFA content and transgene expression in leaves of energycane

Construct	Lines	TAG (% of DW)	TFA (% of DW)	Transgene expressions normalized to *GAPDH*		
				*WRI*1	*DGAT*1	*OLE*1
None	WT	0.02 ± 0.01^a^	1.38 ± 0.02^a^	0.00 ± 0.00^a^	0.00 ± 0.00^a^	0.00 ± 0.00^a^
WDO	L4	0.33 ± 0.05^bc^	2.33 ± 0.05^bcd^	0.00 ± 0.00^a^	0.04 ± 0.01^ab^	0.25 ± 0.10^abcd^
WDO	L5	0.45 ± 0.02^bc^	2.39 ± 0.05^bcd^	0.01 ± 0.00^ab^	0.06 ± 0.00^ab^	0.09 ± 0.01^ab^
WDO	L6	0.56 ± 0.02^bcd^	2.78 ± 0.05^bcde^	0.03 ± 0.01^c^	0.09 ± 0.03^abc^	0.19 ± 0.04^abc^
WDO	L7	0.65 ± 0.01^bcd^	2.96 ± 0.10^bcde^	0.03 ± 0.00^c^	0.10 ± 0.01^abc^	0.31 ± 0.03^bcd^
WDO	L8	1.01 ± 0.01^cde^	3.05 ± 0.12^cde^	0.05 ± 0.01^d^	0.12 ± 0.01^abc^	0.49 ± 0.07^d^
**WDO mean**		**0.60**	**2.70**	**0.02**	**0.08**	**0.27**
WDiO	L9	0.23 ± 0.03^b^	2.06 ± 0.08^ab^	0.01 ± 0.01^ab^	0.01 ± 0.00^a^	0.02 ± 0.00^a^
WDiO	L10	0.28 ± 0.01^bc^	2.15 ± 0.11^abc^	0.00 ± 0.00^a^	0.06 ± 0.03^ab^	0.21 ± 0.05^abc^
WDiO	L11	0.93 ± 0.09^bcde^	3.01 ± 0.05^bcde^	0.02 ± 0.01^bc^	0.79 ± 0.16^d^	1.36 ± 0.13^e^
WDiO	L12	2.68 ± 0.53^f^	5.71 ± 0.64^f^	0.03 ± 0.01^c^	0.92 ± 0.10^d^	1.22 ± 0.24^e^
WDiO	L13	3.85 ± 0.65^ g^	8.39 ± 0.92^ g^	0.03 ± 0.01^c^	1.01 ± 0.21^d^	1.87 ± 0.14^f^
**WDiO mean**		**1.59**	**4.26**	**0.02**	**0.56**	**0.94**

TAG values and gene expression are shown for each line representing leaf extracts from three replications. The leaf samples used are the first dewlap leaf. Values are means ± SE (*n* = 3). The expression of transgenes is shown relative to *GAPDH*. WT indicates the non-transgenic plants. WDO indicates the transgenic lines expressing *WRI*1, *DGAT*1 without intron, *OLE*1 and *npt*II. WDiO indicates the transgenic lines expressing *WRI*1, *DGAT*1 with intron, *OLE*1 and *npt*II. Bold values indicate the mean of all WDO or all WDiO lines, respectively. Values in the same column with different lower case letters are significantly different at* p* ≤ 0.05

**Table 2:** Summary of TAG and TFA content and transgene expression in stems of energycane

Construct	Lines	TAG (% of DW)	TFA (% of DW)	Transgene expressions normalized to *GAPDH*		
				*WRI*1	*DGAT*1	*OLE*1
None	WT	0.02 ± 0.00^a^	0.18 ± 0.01^a^	0.00 ± 0.00^a^	0.00 ± 0.00^a^	0.00 ± 0.00^a^
WDO	L6	0.20 ± 0.01^b^	0.55 ± 0.02^ab^	0.01 ± 0.00^a^	0.04 ± 0.00^ab^	0.07 ± 0.00^ab^
WDO	L7	0.19 ± 0.03^b^	0.91 ± 0.12^abc^	0.01 ± 0.00^a^	0.02 ± 0.00^ab^	0.11 ± 0.01^abc^
WDO	L8	0.51 ± 0.08^bc^	0.98 ± 0.11^bc^	0.04 ± 0.01^bc^	0.08 ± 0.01^ cd^	0.25 ± 0.04^ cd^
**WDO mean**		**0.3**	**0.81**	**0.02**	**0.04**	**0.14**
WDiO	L11	0.76 ± 0.03^bcd^	1.59 ± 0.02^ cd^	0.04 ± 0.00^c^	0.11 ± 0.00^d^	0.21 ± 0.01^bcd^
WDiO	L12	1.14 ± 0.16^d^	2.08 ± 0.22^d^	0.04 ± 0.01^c^	0.06 ± 0.01^bcd^	0.32 ± 0.05^d^
WDiO	L13	1.09 ± 0.28^ cd^	1.79 ± 0.29^d^	0.04 ± 0.01^bc^	0.08 ± 0.02^ cd^	0.25 ± 0.07^ cd^
**WDiO mean**		**1.00**	**1.82**	**0.04**	**0.08**	**0.26**

TAG values and gene expression are shown for each line representing stem extracts from three replications. Values shown for each line represent the mean of mature, mid-mature and immature stem samples from three replications. Values are means ± SE (*n* = 3). The expression of transgenes is shown relative to *GAPDH*. WT indicates the non-transgenic plants. WDO indicates the transgenic lines expressing *WRI*1, *DGAT*1 without intron, *OLE*1 and *npt*II. WDiO indicates the transgenic lines expressing *WRI*1, *DGAT*1 with intron, *OLE*1 and *npt*II. Bold values indicate the mean of all WDO or all WDiO lines, respectively. Values in the same column with different lower case letters are significantly different at* p* ≤ 0.05

As shown in Table 1, 8 out of 10 transgenic lines co-expressed all three lipogenic factors with significant differences in transgene expression between the lines. Two of the transgenic lines (L4; L10, Table 1) expressed *DGAT*1 and *OLE*1 but not *WRI*1. Among the three transgenes, *WRI*1 expression showed the lowest variation ranging from 0.01 to 0.05 relative to *GAPDH* and averaged 0.02 across the five *DGAT*1 (W) or *DGAT*1 (In) lines (Table 1). In WDO combination, TAG accumulated up to 1.01% of leaf DW and 0.22% of stem DW, which was 50-fold and tenfold of that of WT, respectively (Tables 1, 2). The highest TAG accumulation was observed in WDiO combination with up to 3.85% of leaf DW and 1.14% of stem DW, which was 192-fold and 56-fold of that of WT, respectively (Tables 1, 2). Similarly, TFA was the highest in WDiO combination with up to 8.39% of leaf DW and 2.08% of stem DW (Tables 1, 2). In addition, Pearson’s correlation was evaluated for TAG accumulation and lipid gene expression levels in transgenic plants. As shown in Table S2, TAG accumulation exhibited the highest positive correlation with *DGAT*1(W) (r = 0.97) followed by *OLE*1 (W) (r = 0.89), *DGAT*1 (In) (r = 0.88), and *WRI*1 expression (r = 0.51).

### Alteration of FA accumulation in leaves and stem of high TAG transgenic energycane

**Fig. 1:**
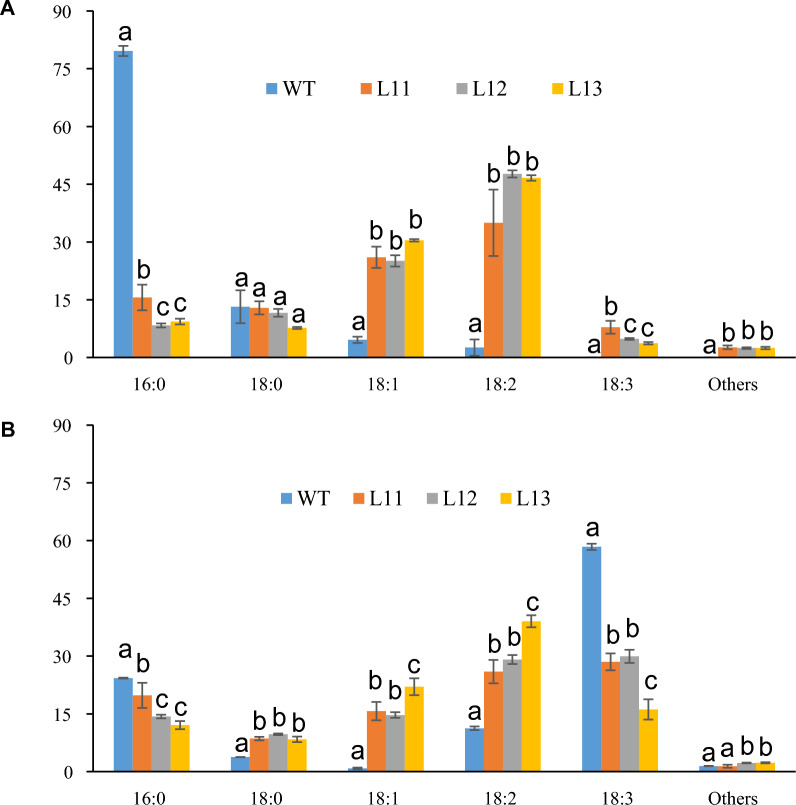
TAG FA composition and total FA composition in leaves of different transgenic energycane lines (L11; L12; L13) compared with non-modified energycane (WT). **A** TAG composition in leaves of different transgenic lines (L11; L12; L13) and non-modified energycane (WT). **B** TFA composition in leaves of different transgenic lines (L11; L12; L13) and WT. Vertical bars are the means ± standard error (*n* = 3). Values with different letters are significantly different at *p* ≤ 0.05 according to a one-way ANOVA test and the Duncan’s multiple range test (MRT). TAG: triacylglycerol. FA: fatty acid. WT: non-modified energycane (WT)

**Fig. 2:**
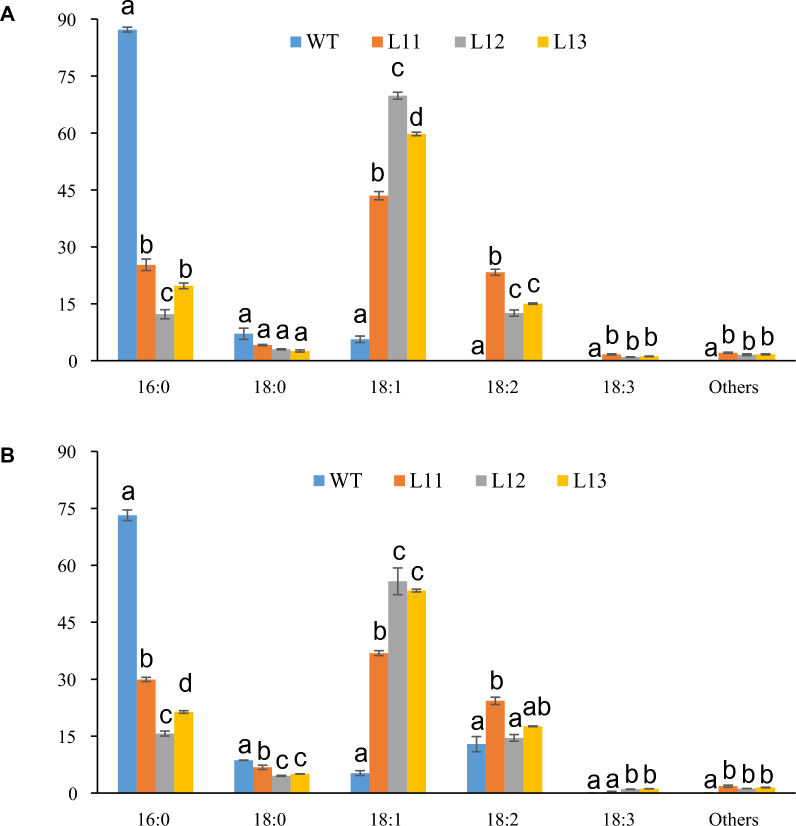
TAG FA composition and total FA composition in stems of different transgenic lines (L11; L12; L13) compared with non-modified energycane (WT). **A** TAG composition in stems of different transgenic lines (L11; L12; L13) and non-modified energycane (WT). **B** TFA compositions in stems of different transgenic lines (L11; L12; L13) and WT. Vertical bars are the means ± standard error (*n* = 3). Values with different letters are significantly different at *p* ≤ 0.05 according to a one-way ANOVA test and the Duncan’s multiple range test (MRT). *TAG* triacylglycerol. *FA* fatty acid. *WT* non-modified energycane (wild-type)

TAG FA composition from leaf (Fig. 1) and the stem (Fig. 2) were analyzed from the three highest oil accumulating lines (L11 – L13). Palmitic acid (PA, 16:0), oleic acid (OA, 18:1), stearic acid (SA, 18:0), linoleic acid (LA, 18:2) and α-linolenic acid (ALA, 18:3) were the major fractions. In both leaf and stem samples (Figs. 1 and 2), levels of unsaturated FA, OA (18:1), and LA (18:2) were significantly increased in the transgenic lines at the expense of saturated FAs, PA (16:0) for TAG in leaves (Fig. 1a) and stems (Fig. 2a), as well as for TFA in leaves (Figs. 1b) and stems (Fig. 2b). Additionally, in the leaf, ALA (18:3) accumulation increased for TAG (Fig. 1a) and was reduced for TFA (Fig. 1b), whereas SA (18:0) accumulation was marginally lowered for TAG (Fig. 1a) and increased for TFA (Fig. 1b). In the stem, the OA (18:1) was the highest for both TAG (Fig. 2a) and TFA (Fig. 2b), in contrast to the FA composition of the leaves, and the SA (18:0) was reduced in both TAG (Fig. 2a) and TFA (Fig. 2b) of the stem. Interestingly, ALA (18:3) was present in transgenic lines but not in wild-type lines (Fig. 2).

### Accumulation of lipid droplets in leaves of transgenic energycane with hyperaccumulation of TAG in comparison to WT controls

**Fig. 3:**
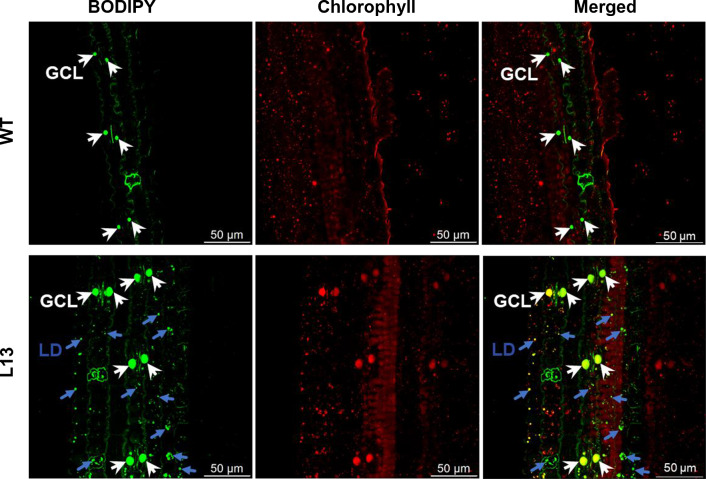
Lipid droplets within the leaf tissue of energecane. The upper panel showcases fluorescence-stained lipid droplets within guard cells (GCL) using BODIPY 493/503, along with the autofluorescence emanating from chloroplasts in the wild-type (WT) strain. The lower panel displays both the lipid droplets within guard cells and those in regular cells (LD), alongside the chloroplast autofluorescence found in leaf tissue of the transgenic line L13. The lipid droplets within guard cells are indicated by white arrows, while a selection of typical lipid droplets are indicated by blue arrows. Both the lipid droplets found within the guard cells (GCL) and those present outside of the guard cells (LD) are rendered in shades of green, while autofluorescence originating from chlorophyll is is seen in red. Scale bar = 50 μm

For the purpose of visualizing lipid droplets, leaves from both the transgenic line L13 and its corresponding WT were subjected to boron-dipyrromethene (BODIPY) staining, followed by imaging using confocal microscopy. Notably, the transgenic line L13, with the overexpression of *DGAT*1 (In), demonstrated a substantial increase in the abundance of BODIPY-stained lipid droplets in comparison to its WT counterpart (Fig. 3). Intriguingly, within the transgenic line L13, the lipid droplets, encompassing both regular lipid droplets (LD) and guard cell lipid droplets (GCL), exhibited a widespread distribution. The most prominent droplet size, around 8 μm in diameter, was predominantly situated within the stomatal guard cells. In contrast, the wild-type leaves exclusively exhibited lipid droplets within stomatal guard cells, typically measuring less than 5 μm in diameter. These findings correlate well with the triacylglycerol (TAG) data depicted in Table 1.

### Impact of hyperaccumulation of TAG on plant growth

To determine the effect of hyperaccumulation of TAG on plant growth, biomass related traits, including plant height, stem diameter, and tiller number were recorded. Hyperaccumulation of TAG corresponded to a reduction in plant height, tiller number, and stem diameter Table 3. Line 13 with the highest TAG accumulation had the greatest reduction in plant height, number of tillers and stem diameter with 31%, 81% and 50%, respectively. In addition, plant height, tiller number and stem diameter correlated negatively with TAG content (*r* = − 0.71, − 0.77 and − 0.50, respectively) (Additional file 2: Table S3).

**Table 3:** Height, stem diameter and number of tillers of the transgenic energycane plants (L11; L12; L13) and non-modified energycane (WT) under greenhouse conditions

Construct	Line	Height (m)	No. of tillers	Stem diameter (cm)
None	WT	1.69 ± 0.16^a^	16 ± 1.80^a^	1.01 ± 0.11^a^
WDiO	L11	1.45 ± 0.17^a^	8 ± 0.66^b^	0.85 ± 0.08^ab^
WDiO	L12	0.99 ± 0.07^b^	4 ± 0.81^c^	0.62 ± 0.09^b^
WDiO	L13	0.89 ± 0.09^b^	3 ± 0.33^c^	0.57 ± 0.08^b^

TAG values and gene expression are shown for each line representing stem extracts from three replications. Values shown for each line represent the mean of mature, mid-mature and immature stem samples from three replications. Values are means ± SE (*n* = 3). The expression of transgenes is shown relative to *GAPDH*. WT indicates the non-transgenic plants. WDiO indicates the transgenic lines expressing *WRI*1, *DGAT*1 with intron, *OLE*1 and *npt*II

## Discussion

Energycane is among the most promising perennial feedstocks to contribute to the emerging bioeconomy due to its high biomass production, yield resilience and ease of biocontainment [4–6, 48]. Metabolic engineering for hyperaccumulation of lipids in vegetative tissues has been proposed for enhancing energy density and biofuel production from high biomass crops like energycane [12, 17, 20, 21]. Metabolic engineering of energycane is still in its infancy due to the recalcitrance of this crop to tissue culture and genetic transformation [17]. Here we report a step change in hyperaccumulation of triacylglycerol (TAG) and total fatty acid (TFA) in energycane using intron-mediated enhancement (IME) of the expression of *DGAT*1. Line-to-line variation in transgene expression in different transgenic events has been reported due to epigenetic changes [49, 50], position of transgene insertion in the genome [51, 52] and copy number of the transgene [53–55]. Therefore, several transgenic events need to be analyzed per recombinant DNA construct to support conclusions on the impact of specific approach/construct. In this study, five lines were evaluated for each, *TmDGAT*1 with and without intron. Including an intron in *TmDGAT*1 (*TmDGAT*1 (In)) increased its expression sevenfold compared to the intron-less variant. Combination of *TmDGAT*1 (In) expression with *SbWRI*1 and *SiOLE*1 expression elevated TAG accumulation 3.8-fold compared to the intron-less combination. Our findings also confirm that *DGAT*1 is a rate-limiting enzyme for TAG accumulation in vegetative tissues of energycane.

The potential of introns to elevate transgene expression by up to 100-fold, termed intron-mediated enhancement (IME) has been described more than 30 years ago [45, 56–61]. IME can strongly stimulate mRNA accumulation far downstream of the transcription start site, even in the absence of a promoter, revealing gaps in our understanding of the molecular mechanisms governing gene expression [62]. Identification of introns that confer IME requires systematic evaluation in transgenic cells since not all introns lead to transcription enhancement [62]. Introns can differ in the presence of enhancers [63, 64] or transcription factor binding sites [65]. In addition, splicing can influence transcription by affecting the phosphorylation state of RNA polymerase II [66]. Intron-dependent gene looping allows physical interaction of the promoter and the terminator regions accelerating mRNA processing events including capping and polyadenylation [67–69]. Disposition of the exon junction complex proteins also enhances mRNA stability, export and translation [70–72].

The impact of introns on transgene expression is influenced by a variety of factors including position of introns [73–75], length of exons [76], specific intronic sequence elements [63, 74, 77], splicing efficiency [74, 76] and their ability to promote the formation of a multi-looped gene architecture [47, 74]. Therefore, not all introns lead to elevated gene expression [78]. Recent studies have identified specific *cis*-elements important for IME expression [46, 79–83]. The intron that was used in this study is constitutively spliced and contains the NGATY core of the longer TTNGATYTG motif and two additional motifs that are similar to TTNGATYTG [46, 83]. These *cis*-elements could play a role in IME, in addition to reduced exon length and supporting transcript processing and nuclear export with the constitutive splicing mechanism. The online transgene design tool “Intronserter”: https://bibiserv.cebitec.uni-bielefeld.de/intronserter supports systematic intron insertion into transgenes for reduction of exon length, along with codon optimization and removal of undesired sequence elements. The program is fully customizable for individual eukaryotic target organisms and should support the systematic design of transgenes for IME in support of metabolic engineering strategies [84].

In the current study, by co-expression of *WRI*1, *DGAT*1 (In)*,* and *OLE*1, we showed an average TAG hyperaccumulation of 3.85% of leaf DW. This TAG content was 192-fold higher than in WT energycane and 2.5-fold higher than reported recently for energycane [17]. The hyperaccumulation of total fatty acid was also elevated to 8.4% of leaf DW which is almost 56 times that of WT and 1.7-fold than in the first report of metabolic engineering of energycane [17]. High level of TAG and TFA accumulation in vegetative tissues of plants typically requires co-expression of several lipogenic factors involved in upregulation of fatty acid biosynthesis, TAG biosynthesis and its protection from degradation [18, 20, 23, 27]. Constitutive co-expression of *WRI*1, Tm*DGAT*1 (In)*,* and *OLE*1 in this study led to 2.5-fold higher TAG and 1.9-fold higher TFA accumulation in energycane than using *ZmDGAT*1-2 without intron and *OLE*1 in combination with RNAi co-suppression of the TAG lipase *sdp*1 and *tgd*1, a lipid transporter from the endoplasmic reticulum (ER) to chloroplasts [17]. We expect the optimization of expression cassettes using IME for all lipogenic factors, codon optimization, stacking of additional lipid genes in combination with gene editing approaches [85–89] will further elevate oil accumulation in energycane.

Reported TFA contents following ectopic co-expression of *WRI*1, *DGAT*1*,* and *OLE*1, in grain sorghum [27] (up to 9.7% of DW) or tobacco [24] (up to 17.7% of DW) exceed the levels of TFA reported here for energycane (8.4% of leaf DW) by 1.16- to 2.11-fold, respectively. However, biomass yield for energycane are typically in the range of 41 to 49 t ha^−1^ in the Southeastern USA [90, 91] which is 3- to 12-fold higher than typical biomass yields from grain sorghum (4 to 12 t ha^−1^) [92, 93] and 3- to 9-fold higher than typical tobacco yields (5.5 to 13 t ha^−1^) [94, 95]. This should lead to higher or similar oil yields from the energycane described here compared to the alternative crops. Biodiesel derived from lipid accumulating energycane is an attractive approach to minimize the use of food and feed crops in the production of renewable transportation fuels. Additional feedstocks and engineering strategies will be needed to establish a robust pipeline for production of sustainable aviation fuel [96].

Coordinating the expression of *WRI*1 with stem specific or developmentally regulated promoters active late in plant development [97, 98] or inducible promoters [43, 99] may mitigate the negative impact on biomass accumulation due to constitutive expression of *WRI*1 and other lipogenic factors described in this and several other studies [18, 25, 27].

The overall TAG and TFA compositions were significantly altered by the co-expression of the lipogenic factors in energycane. Most notable was the increase in oleic acid (18:1) and linoleic acid (18:2) in both, TAG and TFA of transgenic leaves and stems at expense of saturated fatty acids. Similar findings were earlier reported for sugarcane by Parajuli et al. [18]. A significant reduction of alpha linoleic acid (18:3) was only observed in the TFA of leaves. Interestingly in stem tissues oleic acid (18:1) linoleic acid (18:2) and alfa-linolenic acids (18:3) were significantly elevated in both TAG and TFA which differs from the observation in sugarcane using different lipogenic factors [18]. The elevated amount of unsaturated FA in vegetative tissues of energycane should lead to improved fuel stability and cold flow [100].

The presence of leaf GCL droplets as seen in guard cells of WT have been reported for more than three decades [101], where they serve as an energy source to power stomatal opening [102]. Subsequent work on Arabidopsis and the lycophyte Selaginella provide evidence that TAG breakdown is an evolutionarily conserved mechanism in light-induced stomatal opening [103]. In the oil accumulating L13, constitutive expression of *WRI*1, *DGAT*1 and *OLE*1 resulted in a substantial boost in GCL abundance, and LDs were generally visible in surrounding tissue, that were absent in WT controls. The pattern of widespread LD accumulation in transgenic energycane leaves is consistent with reports from transgenic sugarcane [18], tobacco [21], and the duckweed Lemna japonica [43].

## Conclusions

Energycane is a favored feedstock for the production of bioproducts and advanced renewable fuels because of its high biomass production and resilience under marginal conditions. In this study, we demonstrated that *DGAT*1 is the principal rate-limiting step for TAG and TFA accumulation in energycane leaves. IME of *DGAT*1 caused a step change in its expression and its co-expression with *WRI*1 and *OLE*1 elevated TAG or TFA content by 192- or 56-fold relative to levels found in the leaves of unmodified energycane, respectively. These results lay the foundation for the commercial production of biodiesel and other FA derivatives and reveal opportunities for further optimizations of high biomass energycane.

## Materials and methods

### Design and synthesis of the optimized *DGAT*1 gene

For codon optimization, the native *DGAT*1 gene from *Tropaeolum majus* (Gene bank AY084052) was optimized by replacing the preferred codons with synonymous codons of *Sorghum bicolor*, a close relative of sugarcane, without changing the encoded amino acids (Additional file 1: Figure S1). Two optimized *DGAT*1, including *DGAT*1(W) without intron and *DGAT*1 (In) with the 110-bp-long intron from the predicted 5-methyltetrahydropteroyltriglutamate-homocysteine methyltransferase 1 of *Sorghum bicolor* (NCBI Accession number: NC_012877.2), were synthesized by GenScript (Piscataway NJ, USA). The specific intron was selected based on its compact size and the presence of *cis*-regulatory elements that may impact IME (Additional file 1: Figure S1).

### Construction of the multigene expression vectors

**Fig. 4:**
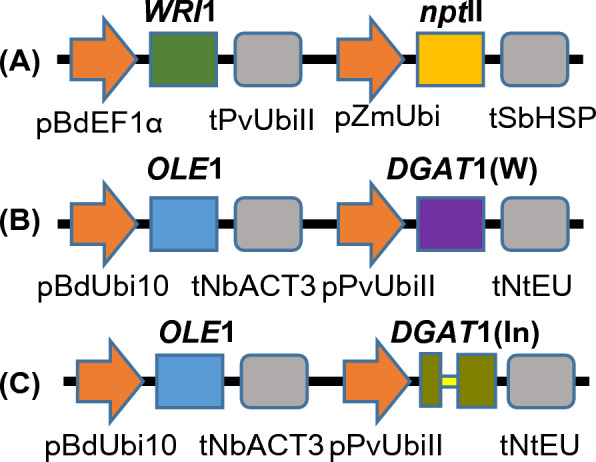
Recombinant DNA constructs used in energy cane transformation. **A** Expression cassettes of *WRI*1 from Sorghum bicolor controlled by the *Brachypodium distachyon* elongation factor 1-α promoter (pBdEF1α) and *Panicum virgatum* ubiquitin (tPvUbiII) terminator, and the selectable marker gene neomycin phosphotransferase (*npt*II) driven by the maize ubiquitin promoter (pZmUbi) and *Sorghum bicolor heat shock protein* terminator (tSbHSP). **B** Expression cassettes for *OLE*1, obtained from *Sesamum indicum*, was under the control of the *Brachypodium distachyon* ubiquitin promoter (pBdUbi10) and *Nicotiana benthamiana* actin 3ʹ UTR terminator (tNbACT3), and *DGAT*1 from *Tropaeolum majus*, without intron was cloned under the control of the *Panicum virgatum* ubiquitin promoter (pPvUbiII) and *Nicotiana tabacum* extensin terminator (tNtEU). **C** Expression cassettes for *OLE*1, obtained from *Sesamum indicum*, was under the control of the *Brachypodium distachyon* ubiquitin promoter (pBdUbi10) and *Nicotiana benthamiana* actin 3ʹ UTR terminator (tNbACT3), and *DGAT*1 from *Tropaeolum majus*, including a 110-bp-long intron from the predicted *Sorghum bicolor* 5-methyltetrahydropteroyltriglutamate-homocysteine methyltransferase 1 was cloned under the control of the *Panicum virgatum* ubiquitin promoter (pPvUbiII) and *Nicotiana tabacum* extensin terminator (tNtEU) W: no intron. In: intron

Two different optimized *DGAT*1 genes obtained from *Tropaeolum majus*, including *DGAT*1(W) without intron and *DGAT*1 (In) with a 110-bp-long intron were compared in this study. Using the Golden Gate cloning strategy [104, 105], three multigene expression vectors were created (Fig. 4). The first vector, shown in Fig. 4A, contains *WRI*1 from *Sorghum bicolor* controlled by the *Brachypodium distachyon elongation factor 1-α* promoter (pBdEF1α) and *Panicum virgatum ubiquitin* (tPvUbiII) terminator, and the selectable marker gene *neomycin phosphotransferase* (*npt*II) driven by the maize *ubiquitin* promoter (pZmUbi) and *Sorghum bicolor heat shock protein* terminator (tSbHSP; Fig. 4A). Vectors shown in Fig. 4B and C contained expression cassettes for two genes: *OLE*1, obtained from *Sesamum indicum,* was under the control of the *Brachypodium distachyon ubiquitin* promoter (pBdUbi10) and *Nicotiana benthamiana actin* 3’ UTR terminator (tNbACT3), and *DGAT*1 from *Tropaeolum majus*, with or without intron was cloned under the control of the *Panicum virgatum ubiquitin* promoter (pPvUbiII) and *Nicotiana tabacum extensin* terminator (tNtEU; Fig. 4B and C).

### Tissue culture and genetic transformation of energycane

**Fig. 5:**
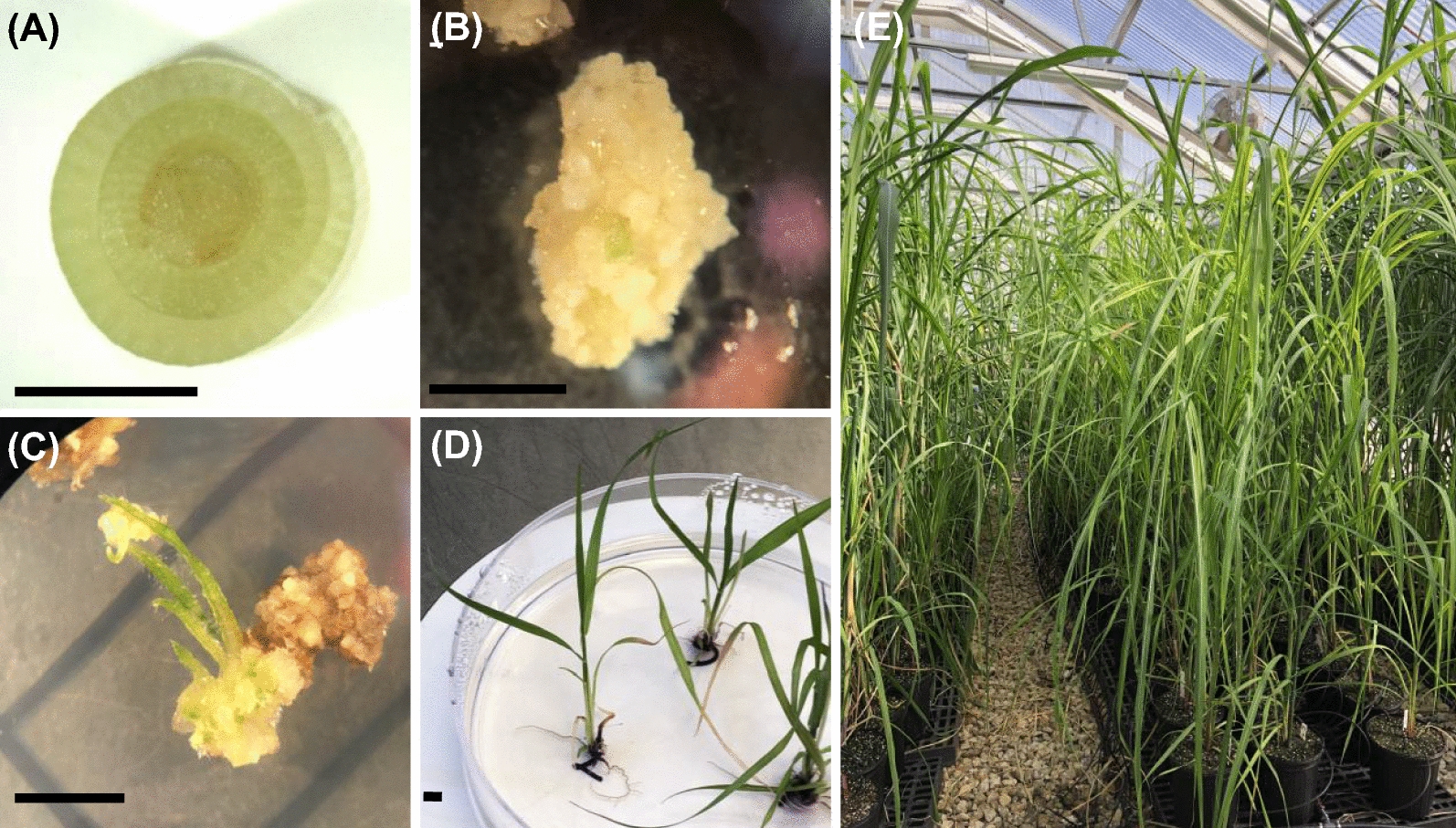
Generation of transgenic energy cane. **A** Leaf whorl cross-sections were used as explants. **B** Callus induced from leaf whorl cross-section was used as the target for biolistic gene transfer. **C** Calli regenerating on medium with geneticin for selection of *npt*II expression. **D** Regenerated plants before transferring to soil. **E** Transgenic energy cane lines growing in greenhouse. Bar = 5 mm

For this study, the energy cane genotype UFCP 84-1047 (WT) was chosen for its superior biomass and resilience [106]. Immature leaf whorl cross-sections (Fig. 5A) were cultured to induce embryogenic callus (Fig. 5B) as described by Fouad et al., [107]. The media for callus induction, transformation, selection, and shoot and root regeneration were prepared according to Fouad et al. [107].

Prior gene transfer, plasmids were digested using the restriction enzyme *Asc*I to remove the backbone. Linearized fragments were gel extracted and purified according to Fouad et al. [107]. Two different combinations including WDO (*WRI*1 + *npt*II: *DGAT*1(W) + *OLE*1), and WDiO (*WRI*1 + *npt*II: *DGAT*1(In) + *OLE*1) were delivered to embryogenic callus by biolistic gene transfer. For each combination, a total of 10 shots of DNA were delivered to callus in a 1:2 molar ratio (*WRI*1 + *npt*II: *DGAT*1 + *OLE*1) using a Biolistic PDS-1000/He particle delivery system (Biorad, Hercules, California, USA) as described earlier [108]. Putative transgenic plants (Fig. 5C, D) were regenerated following selection on geneticin-containing culture medium as described by Fouad et al., 2015 [107]. Rooted plantlets were transferred to soil and grown under greenhouse conditions under natural photoperiod and 25-29ºC day and 20 to 24 °C night temperature (Fig. 5E).

### *Cis*-regulatory elements in the 110-bp intron

PLACE and PlantCARE software were used to evaluate the presence of potential *cis*-regulatory elements in the 110-bp-long intron from the predicted 5-methyltetrahydropteroyltriglutamate-homocysteine methyltransferase 1 of *Sorghum bicolor* (NCBI accession number: NC_012877.2). We also searched for sequence motifs that were earlier reported for their contribution to IME including sequence TTNGATYTG and its reverse complement CAYATCNAA as well as NGATY (or its reverse complement YATCN) [76].

### PCR analysis of genomic DNA extracts

Genomic DNA was extracted from leaves using the cetyl trimethyl ammonium bromide (CTAB) method [109]. 100 ng of genomic DNA was used as a template in a 20-μL PCR reaction. PCR amplification was conducted using Hot Start Taq DNA Polymerase (NEB, Ipswich, Massachusetts, USA) for *WRI*1, *npt*II, *OLE*1, and *DGAT*1 individually following the manufacturer’s protocol. Primers for each target gene are listed in Additional file 2: Table S1. Five transgenic lines in each combination were confirmed by PCR for *WRI*1, *DGAT*1, *OLE*1, and *npt*II (Additional file 1: Figure S2).

### Greenhouse propagation of transgenic, lipid accumulating energycane

In the greenhouse, transgenic energycane (Fig. 5E) was propagated by nodal stem cuttings to obtain biological replicates and three plants per transgenic line and non-transgenic plants were each planted in a pot with a 15 cm diameter containing potting mix (Jolly Gardener C/G, Oldcastle Lawn and Garden, Atlanta GA). Plants were irrigated and fertilized daily with a drip fertigation system. In the greenhouse, the temperature was controlled by evaporation cooling to 25–29 °C during the day and 20–24 °C during the night using natural photoperiod with a maximum daily light intensity of 1000 to 1500 μmol m^−2^ s^−1^.

### Sampling of leaves and stem for quantitative real-time PCR (qRT-PCR), TAG and FA analysis

Leaves from 15 transgenic lines were sliced in half at the midrib; one half was used for qRT-PCR analysis, while the other half was used for TAG and FA analyses. Stem internodes from the three highest TAG lines were also sampled and divided into two halves before grinding in a Retsch cryo bead mill (Verder Scientific, Haan, Germany). For qRT-PCR analysis, samples were flash-frozen in liquid nitrogen and maintained at − 80 °C for later RNA extraction. For TAG and FA analyses, each sample consisted of at least 100 mg of leaf or stem fresh weight and was freeze-dried for 72 h in a lyophilizer (Labconco, MO, USA). For TAG and FA analyses samples were shipped to Brookhaven National Laboratory on dry ice.

### qRT-PCR analysis for transgene expression

TRIzol reagent was used to extract total RNA from leaf/stem samples (Life Technologies, Thermo Fisher Scientific, Waltham, MA, USA). A High Capacity cDNA Reverse Transcription Kit (Applied Biosystems, Foster City, CA, USA) was used to synthesize cDNA from approximately 1.0 µg of total RNA from each sample. *DGAT*1, *OLE*1, and *WRI*1 expressions were measured using primers shown in Additional file 2: Table S1. The glyceraldehyde 3-phosphate dehydrogenase (*GAPDH*) gene was used as a housekeeping gene for transcript normalization [110]. qRT-PCR was performed in a Real-Time PCR Detection System (Biorad, Hercules, CA, USA) using SsoAdvanced Universal SYBR green supermix (Biorad, Hercules, CA, USA). The formula 2^{Ct (*GAPDH*) – Ct (transgene)}^, was used to determine the relative transcription levels of *WRI*1, *DGAT*1, and *OLE*1.

### Analysis of TAG and total FA composition

TAG and FA analyses were conducted according to Zale et al., 2016 [20]. In brief, 10.0 mg of lyophilized leaf/stem tissue was extracted with 700 µL of extraction solution containing 2:1:0.1 by volume methanol:chloroform:formic acid. After 1 h of mixing on a vortex mixer and then standing at RT overnight, extract total lipids were as the lower phase by adding 350 μL of 1 M KCl and 0.2 M H_3_PO_4_ followed by briefly vortexing and centrifuging at 2000 g for 10 min Total lipids were separated by thin layer chromatography (TLC) using a mixture of hexane, diethyl ether and acetic acid (70:30:1, by volume) as mobile phase and TLC Silica gel 60 (EMD Millipore, Cat. No. 1056260001). By spraying 0.05% primuline (in 80% by volume of acetone), lipids were visualized under UV light. TAG fractions were scraped from the TLC plate after identifying by comparing mobility with that of authentic TAG standards. After being incubated in 1.0 mL of boron trichloride–methanol for 40 min at 80–85 °C, TAG fractions were transmethylated into FA methyl esters (FAMEs). FAMES were extracted into hexane and dried under a nitrogen stream before being dissolved in 100 μl hexane and analyzed by GC–MS with an Agilent Technologies (Santa Clara, CA, USA), 7890A GC System equipped with an Agilent 60 m DB23 capillary column (ID 250-μm), and a 5975C mass selective detector in full mass scan mode. The oven temperature was ramped from 100 °C to 240 °C at a rate of 15 °C min^−1^ and held at 240 °C for 3 min with a flow rate of 1.2 ml min^−1^. Following incubation in 1.0 mL of boron trichloride–methanol, total lipid extracts were immediately transmethylated into FAMES for total FA analysis. Using gas chromatography–mass spectrometry, FAMES was dissolved in 100 µL hexane and measured. An internal standard of 5.0 g of C17:0 was employed.

### Visualization of lipid droplets with confocal microscopy

For the visualization of lipid droplets, the third leaf from the non-necrotic tip region of both the transgenic line L13 and its corresponding WT was carefully excised. Subsequently, these leaf samples underwent fixation using a FAA buffer (4% formalin, 5% glacial acetic acid, 50% ethanol, 41% water, V/V) under vacuum conditions for a duration of 1 h. Following fixation, the samples were stained with a solution containing 12 μg/mL of BODIPY 493/503 (Invitrogen, Eugene, OR, USA) and 0.1% Triton X-100, employing vacuum assistance for 20 min. The imaging process was carried out using a Leica TCS SP5 laser scanning confocal microscope, employing an excitation wavelength of 496 nm for BODIPY and capturing emissions within the range of 505–583 nm. Additionally, chlorophyll autofluorescence was captured within the wavelength range of 661–800 nm.

### Analysis of sequence elements

The intron sequence was analyzed in PLACE database (htpp://www.dna.affrc.go.jp/PLACE/signalup.html/, accessed on 14th August) [111] and PlantCARE database (https://bioinformatics.psb.ugent.be/webtools/plantcare/html/, accessed on 14th August 2022) [112].

### Statistical analysis

The TAG content, total FA content, biomass, and qRT-PCR analysis data were expressed as means ± standard error (SE). To conduct statistical analysis of the means, one-way ANOVA was completed using SPSS version 20.0 (Armonk, NY, USA). A value of *p* ≤ 0.05 was considered statistically significant. Pearson’s correlation coefficient was also calculated using SPSS. For each statistical analysis, three independent biological replications were used.

### Supplementary Information

**Additional file 1: Figure S1. Comparison of a native *DGAT*1 gene (I) from *Tropaeolum majus* and an optimized *DGAT*1 gene (II). **The underlined letters indicate nucleotides that were modified for codon optimization. The lowercase letters indicate the inserted intron. The TTNGATYTG-like motif is highlighted in purple with nucleotides that deviated from the motif are marked in green. Extra nucleotide compared to the TTNGATYTG is highlighted in gray. The GT1-consensus, the CAAT box and the NGATY core of the longer TTNGATYTG motif are highlighted in blue, yellow, and dark yellow, respectively. **Figure S2. PCR analysis of transgenic plants. A** to **F**. PCR amplification of *DGAT*1(W), *DGAT*1(In), *OLE*1,* WRI*1, or *npt*II, from genomic DNA of transgenic plants, respectively. PC. Positive control (plasmid used for transformation), Genomic DNA extracts of non-transgenic energycane plant (WT) were used as the negative control. W: no intron. In: intron. Arrows indicate target amplicon.

**Additional file 2: Table S1.** List of primers used for gene expression analysis. **Table S2.** Correlation of total FA content with TAG content in transgenic energycane during plant development. **Table S3.** Correlation of TAG content with total FA, biomass yield DW, height, circumference, stem diameter and tiller number in transgenic energycane.

## Data Availability

All data generated or analyzed during this study are included in this published article (and its Additional information files).
