# Supplementary material for: Intron-mediated enhancement of DIACYLGLYCEROL ACYLTRANSFERASE1 expression in energycane promotes a step change for lipid accumulation in vegetative tissues
Source: Biotechnol Biofuels Bioprod. 2023 Oct 14;16:153. doi: 10.1186/s13068-023-02393-1 (PMC10576891; doi:10.1186/s13068-023-02393-1)
Supplement: Supplementary file 1 — Additional file 1: Figure S1. Comparison of a native DGAT1 gene (I) from Tropaeolum majus and an optimized DGAT1 gene (II). The underlined letters indicate nucleotides that were modified for codon optimization. The lowercase letters indicate the inserted intron. The TTNGATYTG-like motif is highlighted in purple with nucleotides that deviated from the motif are marked in green. Extra nucleotide compared to the TTNGATYTG is highlighted in gray. The GT1-consensus, the CAAT box and the NGATY core of the longer TTNGATYTG motif are highlighted in blue, yellow, and dark yellow, respectively. Figure S2. PCR analysis of transgenic plants. A to F. PCR amplification of DGAT1(W), DGAT1(In), OLE1, WRI1, or nptII, from genomic DNA of transgenic plants, respectively. PC. Positive control (plasmid used for transformation), Genomic DNA extracts of non-transgenic energycane plant (WT) were used as the negative control. W: no intron. In: intron. Arrows indicate target amplicon. [file 13068_2023_2393_MOESM1_ESM.pptx]

## Slide 1
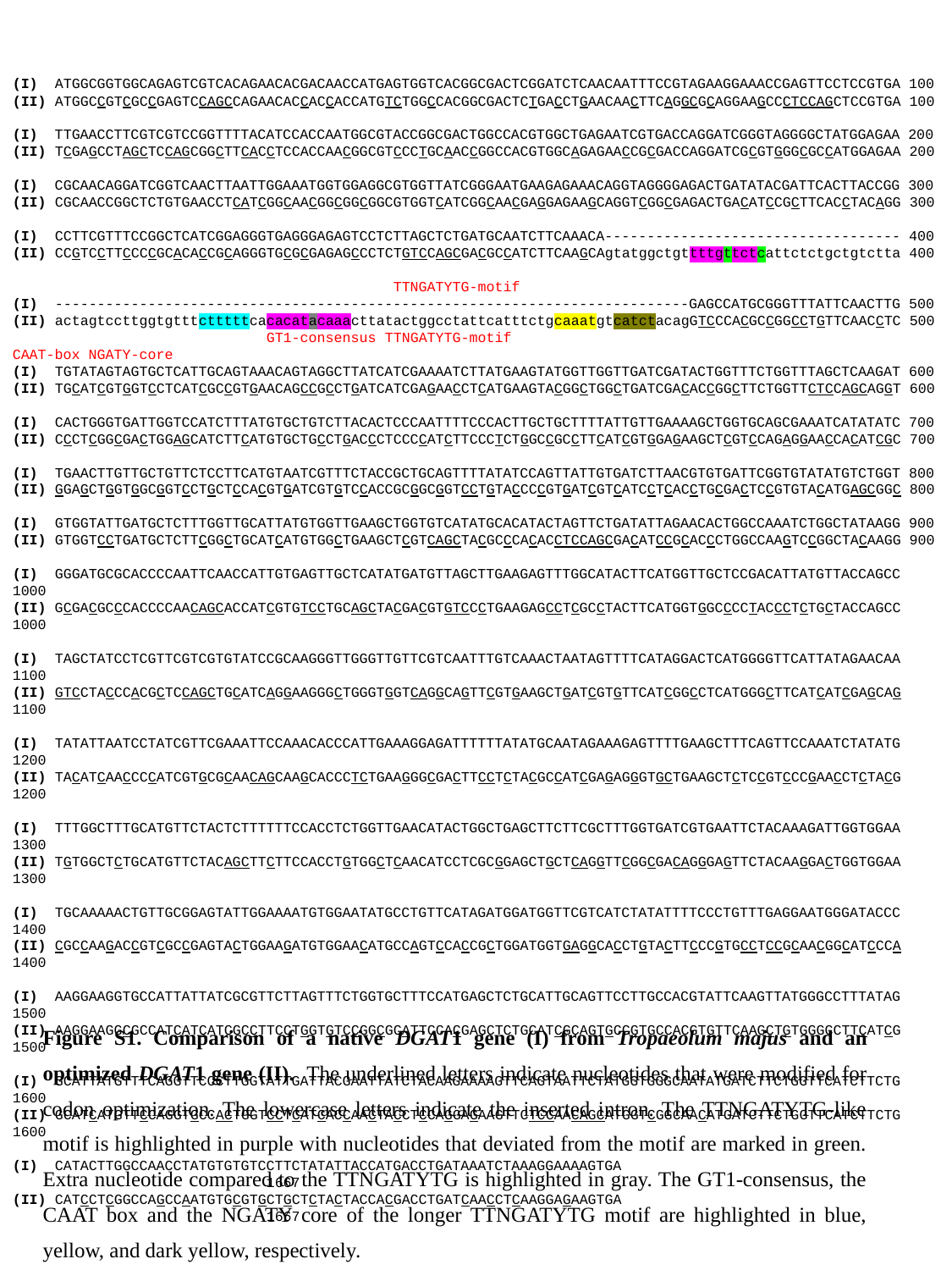

(I) ATGGCGGTGGCAGAGTCGTCACAGAACACGACAACCATGAGTGGTCACGGCGACTCGGATCTCAACAATTTCCGTAGAAGGAAACCGAGTTCCTCCGTGA 100
(II) ATGGCCGTCGCCGAGTCCAGCCAGAACACCACCACCATGTCTGGCCACGGCGACTCTGACCTGAACAACTTCAGGCGCAGGAAGCCCTCCAGCTCCGTGA 100
(I) TTGAACCTTCGTCGTCCGGTTTTACATCCACCAATGGCGTACCGGCGACTGGCCACGTGGCTGAGAATCGTGACCAGGATCGGGTAGGGGCTATGGAGAA 200
(II) TCGAGCCTAGCTCCAGCGGCTTCACCTCCACCAACGGCGTCCCTGCAACCGGCCACGTGGCAGAGAACCGCGACCAGGATCGCGTGGGCGCCATGGAGAA 200
(I) CGCAACAGGATCGGTCAACTTAATTGGAAATGGTGGAGGCGTGGTTATCGGGAATGAAGAGAAACAGGTAGGGGAGACTGATATACGATTCACTTACCGG 300
(II) CGCAACCGGCTCTGTGAACCTCATCGGCAACGGCGGCGGCGTGGTCATCGGCAACGAGGAGAAGCAGGTCGGCGAGACTGACATCCGCTTCACCTACAGG 300
(I) CCTTCGTTTCCGGCTCATCGGAGGGTGAGGGAGAGTCCTCTTAGCTCTGATGCAATCTTCAAACA----------------------------------- 400
(II) CCGTCCTTCCCCGCACACCGCAGGGTGCGCGAGAGCCCTCTGTCCAGCGACGCCATCTTCAAGCAgtatggctgttttgttctcattctctgctgtctta 400
										TTNGATYTG-motif
(I) ---------------------------------------------------------------------------GAGCCATGCGGGTTTATTCAACTTG 500
(II) actagtccttggtgtttctttttcacacatacaaacttatactggcctattcatttctgcaaatgtcatctacagGTCCCACGCCGGCCTGTTCAACCTC 500
		GT1-consensus TTNGATYTG-motif 			 CAAT-box NGATY-core
(I) TGTATAGTAGTGCTCATTGCAGTAAACAGTAGGCTTATCATCGAAAATCTTATGAAGTATGGTTGGTTGATCGATACTGGTTTCTGGTTTAGCTCAAGAT 600
(II) TGCATCGTGGTCCTCATCGCCGTGAACAGCCGCCTGATCATCGAGAACCTCATGAAGTACGGCTGGCTGATCGACACCGGCTTCTGGTTCTCCAGCAGGT 600
(I) CACTGGGTGATTGGTCCATCTTTATGTGCTGTCTTACACTCCCAATTTTCCCACTTGCTGCTTTTATTGTTGAAAAGCTGGTGCAGCGAAATCATATATC 700
(II) CCCTCGGCGACTGGAGCATCTTCATGTGCTGCCTGACCCTCCCCATCTTCCCTCTGGCCGCCTTCATCGTGGAGAAGCTCGTCCAGAGGAACCACATCGC 700
(I) TGAACTTGTTGCTGTTCTCCTTCATGTAATCGTTTCTACCGCTGCAGTTTTATATCCAGTTATTGTGATCTTAACGTGTGATTCGGTGTATATGTCTGGT 800
(II) GGAGCTGGTGGCGGTCCTGCTCCACGTGATCGTGTCCACCGCGGCGGTCCTGTACCCCGTGATCGTCATCCTCACCTGCGACTCCGTGTACATGAGCGGC 800
(I) GTGGTATTGATGCTCTTTGGTTGCATTATGTGGTTGAAGCTGGTGTCATATGCACATACTAGTTCTGATATTAGAACACTGGCCAAATCTGGCTATAAGG 900
(II) GTGGTCCTGATGCTCTTCGGCTGCATCATGTGGCTGAAGCTCGTCAGCTACGCCCACACCTCCAGCGACATCCGCACCCTGGCCAAGTCCGGCTACAAGG 900
(I) GGGATGCGCACCCCAATTCAACCATTGTGAGTTGCTCATATGATGTTAGCTTGAAGAGTTTGGCATACTTCATGGTTGCTCCGACATTATGTTACCAGCC 1000
(II) GCGACGCCCACCCCAACAGCACCATCGTGTCCTGCAGCTACGACGTGTCCCTGAAGAGCCTCGCCTACTTCATGGTGGCCCCTACCCTCTGCTACCAGCC 1000
(I) TAGCTATCCTCGTTCGTCGTGTATCCGCAAGGGTTGGGTTGTTCGTCAATTTGTCAAACTAATAGTTTTCATAGGACTCATGGGGTTCATTATAGAACAA 1100
(II) GTCCTACCCACGCTCCAGCTGCATCAGGAAGGGCTGGGTGGTCAGGCAGTTCGTGAAGCTGATCGTGTTCATCGGCCTCATGGGCTTCATCATCGAGCAG 1100
(I) TATATTAATCCTATCGTTCGAAATTCCAAACACCCATTGAAAGGAGATTTTTTATATGCAATAGAAAGAGTTTTGAAGCTTTCAGTTCCAAATCTATATG 1200
(II) TACATCAACCCCATCGTGCGCAACAGCAAGCACCCTCTGAAGGGCGACTTCCTCTACGCCATCGAGAGGGTGCTGAAGCTCTCCGTCCCGAACCTCTACG 1200
(I) TTTGGCTTTGCATGTTCTACTCTTTTTTCCACCTCTGGTTGAACATACTGGCTGAGCTTCTTCGCTTTGGTGATCGTGAATTCTACAAAGATTGGTGGAA 1300
(II) TGTGGCTCTGCATGTTCTACAGCTTCTTCCACCTGTGGCTCAACATCCTCGCGGAGCTGCTCAGGTTCGGCGACAGGGAGTTCTACAAGGACTGGTGGAA 1300
(I) TGCAAAAACTGTTGCGGAGTATTGGAAAATGTGGAATATGCCTGTTCATAGATGGATGGTTCGTCATCTATATTTTCCCTGTTTGAGGAATGGGATACCC 1400
(II) CGCCAAGACCGTCGCCGAGTACTGGAAGATGTGGAACATGCCAGTCCACCGCTGGATGGTGAGGCACCTGTACTTCCCGTGCCTCCGCAACGGCATCCCA 1400
(I) AAGGAAGGTGCCATTATTATCGCGTTCTTAGTTTCTGGTGCTTTCCATGAGCTCTGCATTGCAGTTCCTTGCCACGTATTCAAGTTATGGGCCTTTATAG 1500
(II) AAGGAAGGCGCCATCATCATCGCCTTCCTGGTGTCCGGCGCATTCCACGAGCTCTGCATCGCAGTGCCGTGCCACGTGTTCAAGCTGTGGGCCTTCATCG 1500
(I) GCATTATGTTTCAGGTTCCCTTGGTATTGATTACGAATTATCTACAAGAAAAGTTCAGTAATTCTATGGTGGGCAATATGATCTTCTGGTTCATCTTCTG 1600
(II) GCATCATGTTCCAGGTGCCACTGGTCCTCATCACCAACTACCTCCAGGAGAAGTTCTCCAACAGCATGGTCGGCAACATGATCTTCTGGTTCATCTTCTG 1600
(I) CATACTTGGCCAACCTATGTGTGTCCTTCTATATTACCATGACCTGATAAATCTAAAGGAAAAGTGA					1667
(II) CATCCTCGGCCAGCCAATGTGCGTGCTGCTCTACTACCACGACCTGATCAACCTCAAGGAGAAGTGA					1667
Figure S1. Comparison of a native DGAT1 gene (I) from Tropaeolum majus and an optimized DGAT1 gene (II). The underlined letters indicate nucleotides that were modified for codon optimization. The lowercase letters indicate the inserted intron. The TTNGATYTG-like motif is highlighted in purple with nucleotides that deviated from the motif are marked in green. Extra nucleotide compared to the TTNGATYTG is highlighted in gray. The GT1-consensus, the CAAT box and the NGATY core of the longer TTNGATYTG motif are highlighted in blue, yellow, and dark yellow, respectively.

## Slide 2
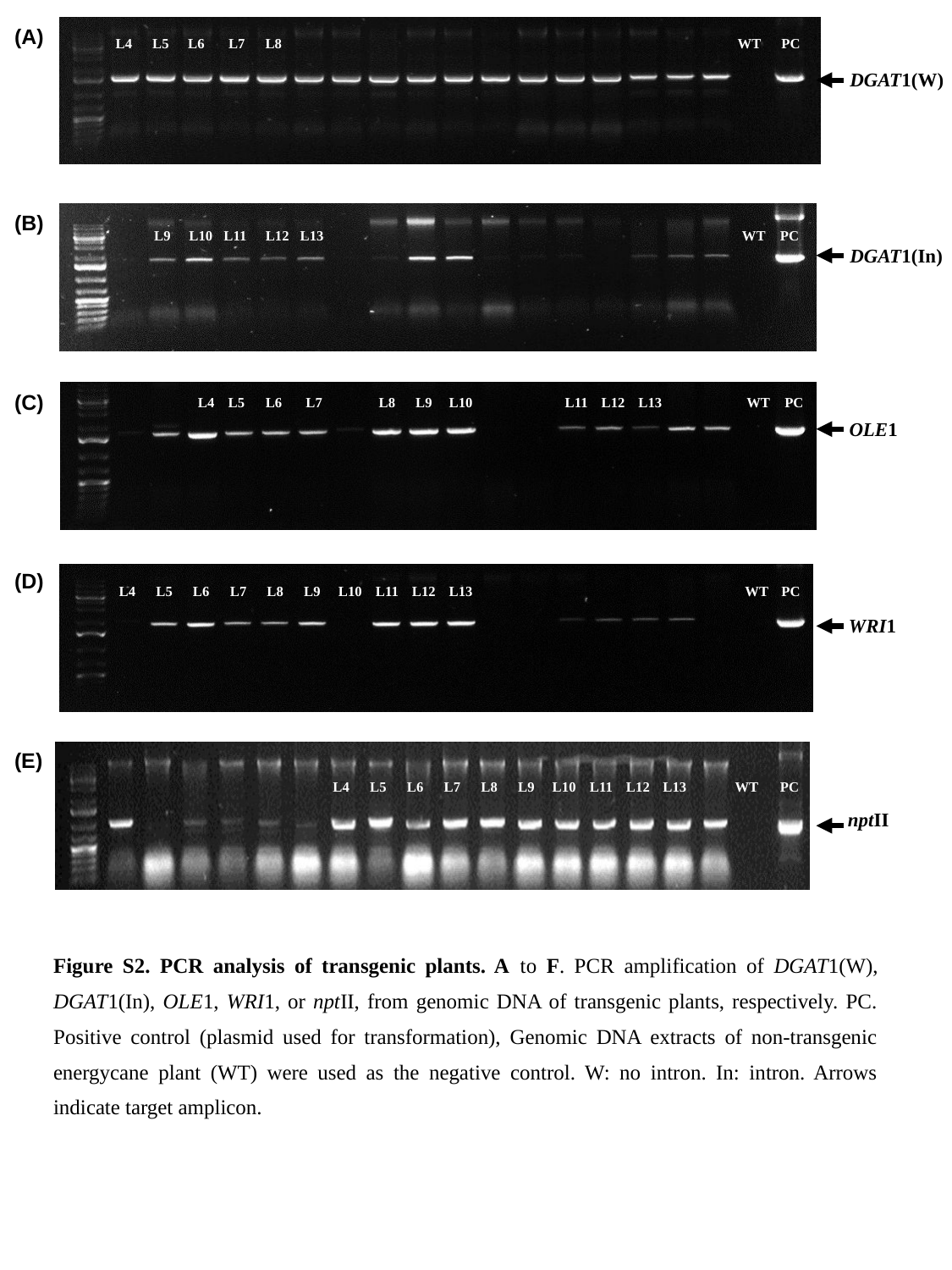

(A)
L4
L5
L6
L7
L8
WT
PC
DGAT1(W)
(B)
L9
L10
L11
L12
L13
WT
PC
DGAT1(In)
(C)
L4
L5
L6
L7
L8
L9
L10
L11
L12
L13
WT
PC
OLE1
(D)
L4
L5
L6
L7
L8
L9
L10
L11
L12
L13
WT
PC
WRI1
(E)
L4
L5
L6
L7
L8
L9
L10
L11
L12
L13
WT
PC
nptII
Figure S2. PCR analysis of transgenic plants. A to F. PCR amplification of DGAT1(W), DGAT1(In), OLE1, WRI1, or nptII, from genomic DNA of transgenic plants, respectively. PC. Positive control (plasmid used for transformation), Genomic DNA extracts of non-transgenic energycane plant (WT) were used as the negative control. W: no intron. In: intron. Arrows indicate target amplicon.
