# Supplementary material for: Intron-mediated enhancement of DIACYLGLYCEROL ACYLTRANSFERASE1 expression in energycane promotes a step change for lipid accumulation in vegetative tissues
Source: Biotechnol Biofuels Bioprod. 2023 Oct 14;16:153. doi: 10.1186/s13068-023-02393-1 (PMC10576891; doi:10.1186/s13068-023-02393-1)
Supplement: Supplementary file 2 — Additional file 2: Table S1. List of primers used for gene expression analysis. Table S2. Correlation of total FA content with TAG content in transgenic energycane during plant development. Table S3. Correlation of TAG content with total FA, biomass yield DW, height, circumference, stem diameter and tiller number in transgenic energycane. [file 13068_2023_2393_MOESM2_ESM.docx]

**Additional tables**

Table S1. List of primers used for gene expression analysis.

Table S2. Correlation of total FA content with TAG content in transgenic energycane during plant development.

Table S3. Correlation of TAG content with total FA, biomass yield DW, height, circumference, stem diameter and tiller number in transgenic energycane.

**Table S1. List of primers used for PCR and gene expression analysis.**

| **Primer name** | **Sequence (5’ – 3’)** | **Amplicon length (bp)** | **Usage** |
| --- | --- | --- | --- |
| pBdUbi10-F | CATGGTTTGCCAGAGTAGTTTG | 1,622 | PCR for *OLE*1 |
| OLE1-R | CTGTAGATCCAGCTGAGGAC |  |  |
| pPvUbiII-F | TCAGCTAGGTCTGCCCTGTT | 1,737 (no intron)  1,847 (with intron) | PCR for *DGAT*1 |
| DGAT1-R | GAACACGATCAGCTTCACGA |  |  |
| pBdEF1α-F | GGCATATCATCCACACGATG | 1,380 | PCR for *WRI*1 |
| WRI1_R | GGGAAGTTGAGCAGAGTC |  |  |
| NPTII-F | AGACAATCGGCTGCTCTGAT | 986 | PCR for *npt*II |
| 35S_NPTII-R | GGTAATGGGGGATCTGGATT |  |  |
| RT_GAPDH-F | CACGGCCACTGGAAGCA | 152 | qRT-PCR for *GAPDH* |
| RT_GAPDH-R | TCCTCAGGGTTCCTGATGCC |  |  |
| RT_DGAT1-F | ATCATCGAGCAGTACATCAA | 145 | qRT-PCR for *DGAT*1 |
| RT_DGAT1-R | GGTGGAAGAAGCTGTAGAAC |  |  |
| RT_OLE1-F | CTGCTCGTGATCTTCTCC | 125 | qRT-PCR for *OLE*1 |
| RT_OLE1-R | CTGTAGATCCAGCTGAGGAC |  |  |
| RT_WRI1-F | CTCCACAACAAGAAGAAGG | 125 | qRT-PCR for *WRI*1 |
| RT_WRI1-R | GGGAAGTTGAGCAGAGTC |  |  |
| RT_NPTII-F | AGACAATCGGCTGCTCTGAT | 156 | qRT-PCR for *npt*II |
| RT_NPTII-R | CTGTGCTCGACGTTGTCACT |  |  |

Note: *DGAT*1: *Diacylglycerol acyltransferase*1; *GAPDH*: *Glyceraldehyde 3-phosphate dehydrogenase*; *WRI*1: *WRINKLED*1; *npt*II: *Neomycin phosphotransferase* II; *OLE*1: *Oleosin*1; Ubi: Ubiquitin; EF1α: Elongation factor 1α; F: forward primer. R: Reverse primer.

**Table S2. Correlation of TAG content with total FA content, and transgene expression in transgenic energycane.**

|  | **TAG** | **TFA** | ***WRI*1** | ***DGAT*1(W)** | ***DGAT*1(In)** | ***OLE*1** |
| --- | --- | --- | --- | --- | --- | --- |
| **TAG** | 1 |  |  |  |  |  |
| **TFA** | 0.99*** | 1 |  |  |  |  |
| ***WRI*1** | 0.51 | 0.49 | 1 |  |  |  |
| ***DGAT*1(W)** | 0.97** | 0.97** | 0.93** | 1 |  |  |
| ***DGAT*1(In)** | 0.88* | 0.86* | 0.95** | - | 1 |  |
| ***OLE*1** | 0.89** | 0.88** | 0.44 | 0.83* | 0.98** | 1 |

Significance in correlation was evaluated with the two-tailed test of significance. * Correlation is significant at the 0.05 level (p ≤ 0.05); ** Correlation is highly significant at the 0.01 level (p ≤ 0.01); n=3. W: no intron. In: intron.

**Table S3. Correlation of TAG content with total FA content, height, tiller number and stem diameter in transgenic energycane.**

|  | **TAG** | **TFA** | **Height** | **Tiller Number** | **Stem diameter** |
| --- | --- | --- | --- | --- | --- |
| **TAG** | 1 |  |  |  |  |
| **TFA** | 0.99** | 1 |  |  |  |
| **Height** | -0.71* | -0.74** | 1 |  |  |
| **Tiller Number** | -0.77** | -0.77** | 0.91** | 1 |  |
| **Stem diameter** | -0.50 | -0.55 | 0.95** | 0.89** | 1 |

Significance in correlation was detected according to two-tailed test of significance. * Correlation is significant at the 0.05 level (p ≤ 0.05); ** Correlation is highly significant at the 0.01 level (p ≤ 0.01); n=3.
